# Endoscopic and clinicopathological features of early gastric papillary adenocarcinoma

**DOI:** 10.3389/fonc.2024.1456520

**Published:** 2024-10-30

**Authors:** Zhenxiang Zuo, Xing Qi, Xiujie Cui, Bin Yu, Huimin Zhang, Honglei Wu

**Affiliations:** ^1^ Department of Gastroenterology, the Second Hospital, Cheeloo College of Medicine, Shandong University, Jinan, China; ^2^ Department of Pathology, the Second Hospital, Cheeloo College of Medicine, Shandong University, Jinan, China

**Keywords:** gastric papillary adenocarcinoma, endoscopic and clinicopathological features, VEC, Mucin, malignant degree

## Abstract

**Objectives:**

Gastric papillary adenocarcinoma (GPA), a well-differentiated gastric adenocarcinoma, is associated with a worse prognosis compared to other differentiated gastric adenocarcinomas. Therefore, there is an urgent need to characterize its endoscopic manifestations for guiding biopsy site selection and achieving accurate diagnosis.

**Methods:**

From January 1, 2016, to December 31, 2022, the data of 46 cases of early gastric papillary adenocarcinoma (EGPA) and 183 cases of early gastric differentiated tubular adenocarcinoma (EGDTA) diagnosed via pathological examination following endoscopic submucosal dissection (ESD) at the Second Hospital of Shandong University were collected. Propensity score matching (PSM) was employed to match 92 EGDTA patients at a ratio of 1:2, serving as the control group. Differences between the two groups were analyzed using multivariable logistic regression. Lastly, the relationship between vessels within epithelial circle (VEC) structures in EGPA and the degree of malignancy was assessed.

**Results:**

Compared with EGDTA, EGPA was more likely to infiltrate the submucosa, more frequently associated with poorly differentiated cancer components, and more prone to invading lymphatic and blood vessels. EGPA was primarily located in the lower stomach and manifested as a uniformly elevated pattern under endoscopy, while VEC structural positivity could be visualized under ME-NBI. Moreover, EGPA lesions had larger diameters and were characterized by high expression of gastric mucins, namely MUC5AC and MUC6. When EGPA infiltrated the submucosa or contained poorly differentiated cancer components, the VEC structures were smaller.

**Conclusions:**

The present study demonstrated that EGPA exhibits a higher degree of malignancy. Endoscopic findings of a raised lesion with a uniform color under endoscopy and the presence of VEC structures under ME-NBI suggest a high possibility of EGPA. Moreover, smaller VEC structures were associated with a higher degree of malignancy, which may assist in guiding the selection of biopsy sites under endoscopy.

## 1 Introduction

Gastric cancer (GC) refers to malignant tumors originating from the epithelial cells of the gastric mucosa. Notably, it ranks fifth in terms of global incidence and cancer-related mortality, making it one of the major public health issues worldwide (1). Early gastric cancer (EGC) often presents with subtle symptoms. Thus, the majority of patients are diagnosed at advanced stages of the disease (2). Treatment options for advanced-stage gastric cancer are limited, with a postoperative 5-year survival rate of approximately 30% (3). The early stage of gastric cancer refers to the stage wherein the cancerous tissue is limited to the mucosal layer or submucosal layer, regardless of lymph node metastasis (LNM) (4). In recent years, the popularization of endoscopic examinations has progressively increased the diagnostic rate of early gastric cancer, while the survival rate of early gastric cancer treated with endoscopic submucosal dissection (ESD) can reach 90.9%-100% (5). In the clinical setting, the combination of gastroscopy biopsy and pathological diagnosis remains the gold standard for the diagnosis of gastric cancer. Early gastric cancers of different differentiation types have distinct white light endoscopic features. However, preoperative biopsy diagnosis and postoperative pathological diagnosis may differ due to limitations in the field of view and biopsy sampling range during gastroscopy. According to a previous study, the combination of magnifying endoscopy with narrow-band imaging (ME-NBI) technology can enhance the accuracy of endoscopic diagnosis for early gastric cancer (6). Therefore, there is a pressing need to explore the characteristic manifestations of early gastric cancer under white light endoscopy and ME-NBI to guide accurate biopsy sampling.

**Figure 1:**
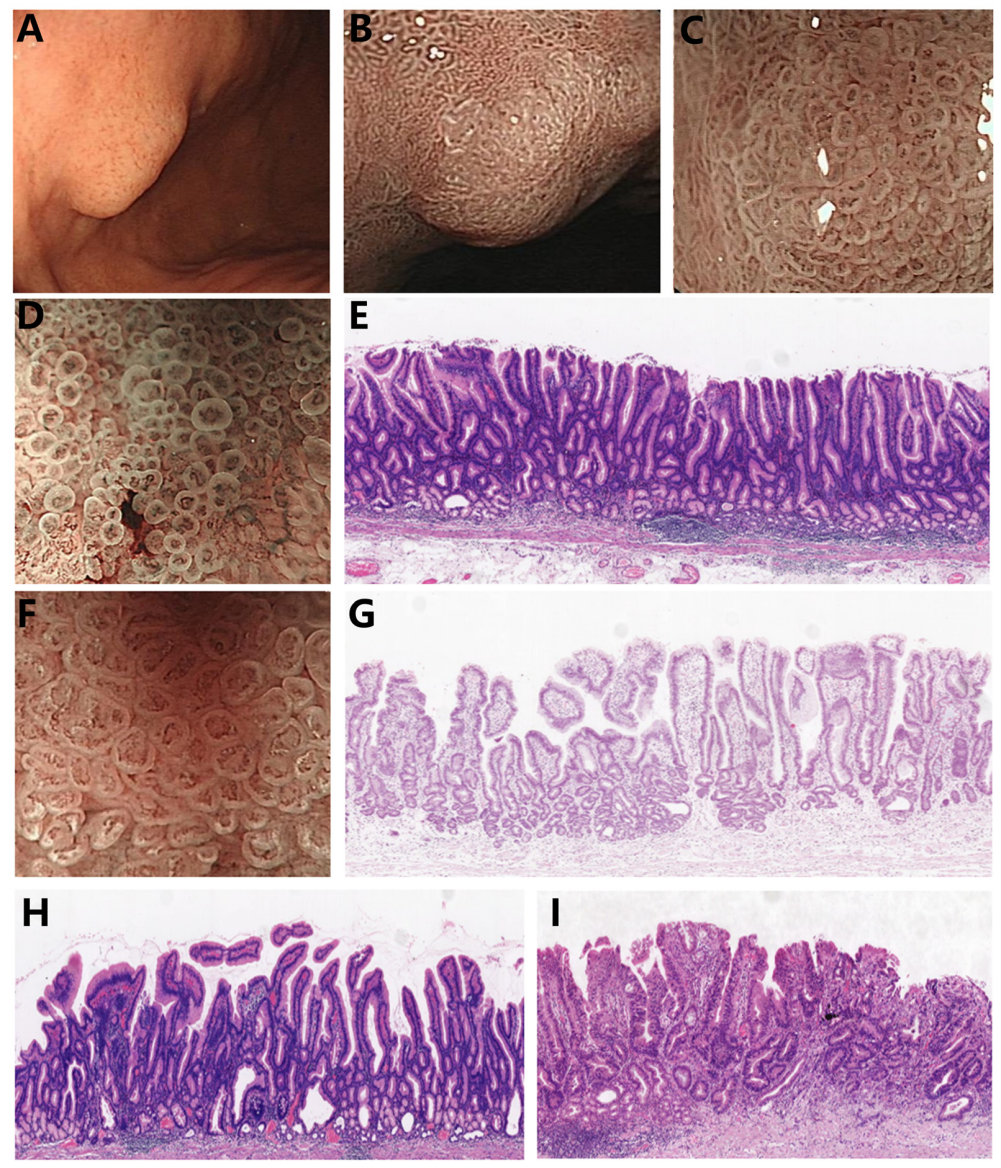
Endoscopic features and pathological characteristics of EGPA **(A)** EGPA presents as a homogeneous color and elevated type under white light endoscopy. **(B, C)** EGPA demonstrates varying performances under ME-NBI at different magnification levels, with the VEC structure visible at high magnification. **(D)** Smaller VEC structure under ME-NBI. **(E)** Corresponding to the narrow nipple structure of the small VEC illustrated in **(D)**. **(F)** Larger VEC structure under ME-NBI. **(G)** Corresponding to the wide nipple structure of the large VEC in **(F)**. **(H)** The EGPA branch is loosely connected. **(I)** EGPA combines poorly differentiated cancer components.

Gastric papillary adenocarcinoma (GPA), a well-differentiated type of differentiated gastric adenocarcinoma, contains tubular components with papillary carcinoma glands. It is histopathologically characterized by finger-like protrusions with a fibrovascular axis and covered by columnar or cuboidal epithelial cells (7). Earlier studies reported that the rates of lymph node metastasis and vascular invasion are higher in differentiated gastric adenocarcinoma with papillary adenocarcinoma glandular structures compared to differentiated gastric adenocarcinoma without papillary adenocarcinoma glandular structures, with patients exhibiting a worse prognosis (8–10). The identification of endoscopic features specific to gastric papillary adenocarcinoma is crucial for facilitating early diagnosis. However, early gastric papillary adenocarcinoma (EGPA) has a low incidence rate (7), and the proportion of papillary adenocarcinoma glandular structures in cancer tissues is similarly low. Thus, routine biopsy sampling sites are linked to an increased misdiagnosis rate. Research has concluded that ME-NBI can visualize a circular structure of blood vessels surrounded by marginal crypt epithelium, referred to as vessels within an epithelial circle (VEC), which is a characteristic endoscopic feature of gastric papillary adenocarcinoma (11) and plays a vital role in biopsy site selection (**Figure 1**). Nevertheless, this finding warrants further validation from multicenter studies. The findings of the current study revealed that the size of VEC structures may be correlated with the malignancy degree of EGPA. Therefore, elucidating the characteristic endoscopic manifestations and pathological features of EGPA and exploring the relationship between VEC structures and the malignancy degree of EGPA hold significant implications in enhancing the diagnostic accuracy of endoscopic and pathological diagnosis, as well as assist in formulating personalized treatment strategies.

Mucin, an extensively glycosylated protein, exhibits remarkably conserved molecular structures across various types of epithelial cells. It plays pivotal roles in diverse physiological processes and serves as a vital protective agent for the body. Moreover, mucin participates in the formation of the gastric mucus barrier and protects the integrity of the gastric mucosal epithelium. In addition, mucins play a decisive role in cellular signaling by interacting with specific receptors on neighboring cells or pathogens, which, in turn, can trigger various cellular responses involved in immune defense responses or tissue repair processes. Previous studies have established that abnormal expression of mucin proteins is involved in the occurrence and invasion of tumors (12). The expression of mucin proteins is tissue-specific. For instance, MUC5AC is primarily secreted by gastric pit epithelial cells and neck mucous cells, MUC6 is largely expressed in the cytoplasm of gastric pyloric glands, and MUC2 is not expressed or minimally expressed in healthy gastric mucosa. However, MUC2 expression is upregulated during gastric mucosal intestinal metaplasia (13, 14). As is well documented, the expression of these three mucin proteins is dysregulated in early gastric cancer (15, 16). According to the expression pattern of mucin proteins, gastric cancer can be classified into gastric, intestinal, mixed gastric-intestinal, and non-mucinous subtypes (17). Clinically, gastric papillary adenocarcinoma typically overexpresses the gastric phenotype mucins MUC5AC and MUC6, which may aid in distinguishing between EGDTA and EGPA. The relationship between gastric mucin expression and the malignancy of EGPA deserves further exploration.

## 2 Methods

Experiments involving humans were approved by the Second Hospital of Shandong University and conducted in accordance with local legislation and institutional requirements. Written informed consent for participation from participants or the participants’ legal guardians/next of kin was waived in accordance with the national legislation and institutional requirements.

### 2.1 Patients

The clinical data of cases who underwent gastric ESD between 2016 and 2022, with pathological diagnoses confirmation of EGPA, were collected. Early gastric differentiated tubular adenocarcinoma (EGDTA) cases were included as the control group. Histopathological classifications were strictly confirmed according to the fifth edition of WHO diagnostic criteria. Cases with a history of partial gastrectomy, as well as prior treatment with radiotherapy/chemotherapy before ESD surgery, were excluded. In instances of multiple ESD or surgical treatments, only cases involving the first treatment and where postoperative pathological specimens were retained at our hospital were included. Eventually, a total of 46 EGPA cases and 183 EGDTA cases were enrolled in the study. Using age as a confounding factor, propensity score matching was performed in a 1:2 ratio to match 92 EGDTA cases as the control group. All cases were from the Second Hospital of Shandong University.

### 2.2 Endoscopic and pathological parameters

The sliced samples were acquired from the pathology department, while endoscopic images were obtained from the digestive endoscopy unit. Data collected encompassed patient age, gender, lesion location, gross type, color tone, VEC structure, presence of ulceration, tumor diameter, depth of infiltration, infiltration pattern, presence of poorly differentiated carcinoma components, and evidence of vascular invasion. According to the third edition of the Japanese gastric cancer classification (18), tumors were classified as upper stomach, middle stomach, and lower stomach. The Paris classification system was applied, and tumors were categorized as 0-I (elevated type), 0-II (superficial type), and 0-III (depressed type). 0-II was further divided into 0-IIa (superficial elevated type), 0-IIb (superficial flat type), and 0-IIc (superficial depressed type). Gross tumor types were classified as elevated (0-I and 0-IIa) and non-elevated (0-IIb, 0-IIc, and 0-III). The infiltration depth of tumors was divided into two types, namely mucosal carcinoma, where the tumor is limited to the mucosal layer or partially invades the mucosal muscular layer, and submucosal carcinoma, where the tumor infiltrates the mucosal muscular layer and reaches the submucosa. Endoscopic images were matched with the corresponding pathological slide locations, using glandular interspace to assess the size of VEC structures.

### 2.3 Immunohistochemical staining

All pathological specimens were sectioned into slices with a thickness of 4 μm and subjected to HE staining and immunohistochemical staining. The pathological diagnosis was collaboratively conducted by three or more experienced pathologists using a multi-headed microscope. Antibodies MUC2, MUC5AC, and MUC6 were purchased from Beijing Zhongshan Golden Bridge Biotechnology Co., Ltd, with positive staining located in the cytoplasm. The scoring criteria for staining intensity ranged from 0 to 3: 0 - no staining, 1 - mild staining, 2 - moderate staining, and 3 - strong staining. The percentage of positive cells was scored on a scale from 0 to 4: 0: 0%, 1: 1%-25%, 2: 26%-50%, 3: 51%-75%, and 4: 76%-100%. The comprehensive score, representing overall expression, was obtained by multiplying the staining intensity score with the percentage of immune reactive cells and ranged between 0 and 12.

### 2.4 Statistical analysis

Statistical analysis were performed utilizing the SPSS 27.0 statistical software. Measurement data following a normal distribution with homogeneity of variance were presented as mean ± standard deviation ( $\bar{\text{x}}$ ± s), and the independent sample t-test was used for between-group comparisons. Non-normally distributed data were expressed as the median (M) and interquartile range (IQR), and the Mann-Whitney U test was used for between-group comparisons. Count data were expressed as frequency, and Pearson’s chi-square test, adjusted Pearson’s chi-square test, or Fisher’s exact test was employed for intergroup comparisons. The R language was utilized for the implementation of propensity score matching. Logistic binary regression analysis was applied to identify independent risk factors for dichotomous variables and only variables with statistical significance were incorporated in univariate analysis. The area under the receiver operating characteristic (ROC) curve (AUC) was used to assess the diagnostic value of risk factors. *P*<0.05 was considered statistically significant.

## 3 Results

### 3.1 Comparative analysis of endoscopic and clinicopathological characteristics between EGPA and EGDTA

As listed in **Table 1**, the 46 patients included 35 males (76.1%) and 11 females (23.9%). EGPA was located in the upper stomach, middle stomach, and lower stomach in 4 (8.7%), 12 (26.1%), and 30 cases (65.2%), respectively. Among them, 32 cases (69.6%) were classified as elevated types (0-I/IIa types), while the remaining 14 cases (30.4%) were categorized as non-elevated types (0-IIb/IIc/III types). Meanwhile, the color tones of the lesions under endoscopic examination were as follows: 15 cases (32.6%) displayed a red tone, 28 cases (60.9%) showed an equal tone, and 3 cases (6.5%) exhibited a faded tone. Moreover, positive and negative VEC structures were identified in 43 (93.5%) and 3 cases (6.5%), respectively. Lastly, ulceration was observed in 2 cases of EGPA (4.3%) under endoscopy, whereas 44 EGPA cases (95.7%) displayed no signs of ulceration.

**Table 1:** Comparative analysis of differences in endoscopic and clinical pathological features between EGPA and EGDTA.

Features	EGPA (n = 46)	EGDTA (n = 92)	χ²/Z/*t*	*P*
Gender, n (%)			*χ²*=0.02	0.889
Male	35 (76.1)	69 (75.0)		
Female	11 (23.9)	23 (25.0)		
Tumor location, n (%)			*χ²*=6.39	0.042
Upper	4 (8.7)	19 (20.6)		
Middle	12 (26.1)	33 (35.9)		
Lower	30 (65.2)	40 (43.5)		
General type, n (%)			*χ²*=26.83	<0.001
Elevated type	32 (69.6)	22 (23.9)		
Non-elevated type	14 (30.4)	70 (76.1)		
Color, n (%)			*χ²*=47.20	<0.001
Red	15 (32.6)	80 (87.0)		
Equal	28 (60.9)	7 (7.6)		
Faded	3 (6.5)	5 (5.4)		
VEC, n (%)			*χ²*=104.79	<0.001
Positive	43 (93.5)	5 (5.4)		
Negative	3 (6.5)	87 (94.6)		
Ulceration, n (%)			–	0.258
Positive	2 (4.3)	1 (1.1)		
Negative	44 (95.7)	91 (98.9)		
Tumor diameter, M (Q₁, Q₃)	2.25 (1.60- 3.00)	1.40 (1.00- 1.98)	*Z*=4.07	<0.001
Low differentiation cancer component, n (%)			–	<0.001
Positive	11 (23.9)	3 (3.3)		
Negative	35 (76.1)	89 (96.7)		
Infiltration pattern, n (%)			–	0.180
INFa	40 (87.0)	87 (94.6)		
INFb/INFc	6 (13.0)	5 (5.4)		
Infiltration depth, n (%)			–	0.007
Mucosa	38 (82.6)	89 (96.7)		
Submucosa	8(17.4)	3 (3.3)		
Lymphovascular invasion, n (%)			–	0.011
Negative	42 (91.3)	92 (100.0)		
Positive	4 (8.7)	0 (0.0)		
MUC2	3 (1.75, 6)	3 (2, 3)	*Z*=1.32	0.186
MUC5AC	6 (6, 12)	2 (1, 4)	*Z*=6.89	<0.001
MUC6	6 (2.75, 8.25)	3 (2, 4)	*Z*=3.92	<0.001

**Figure 2:**
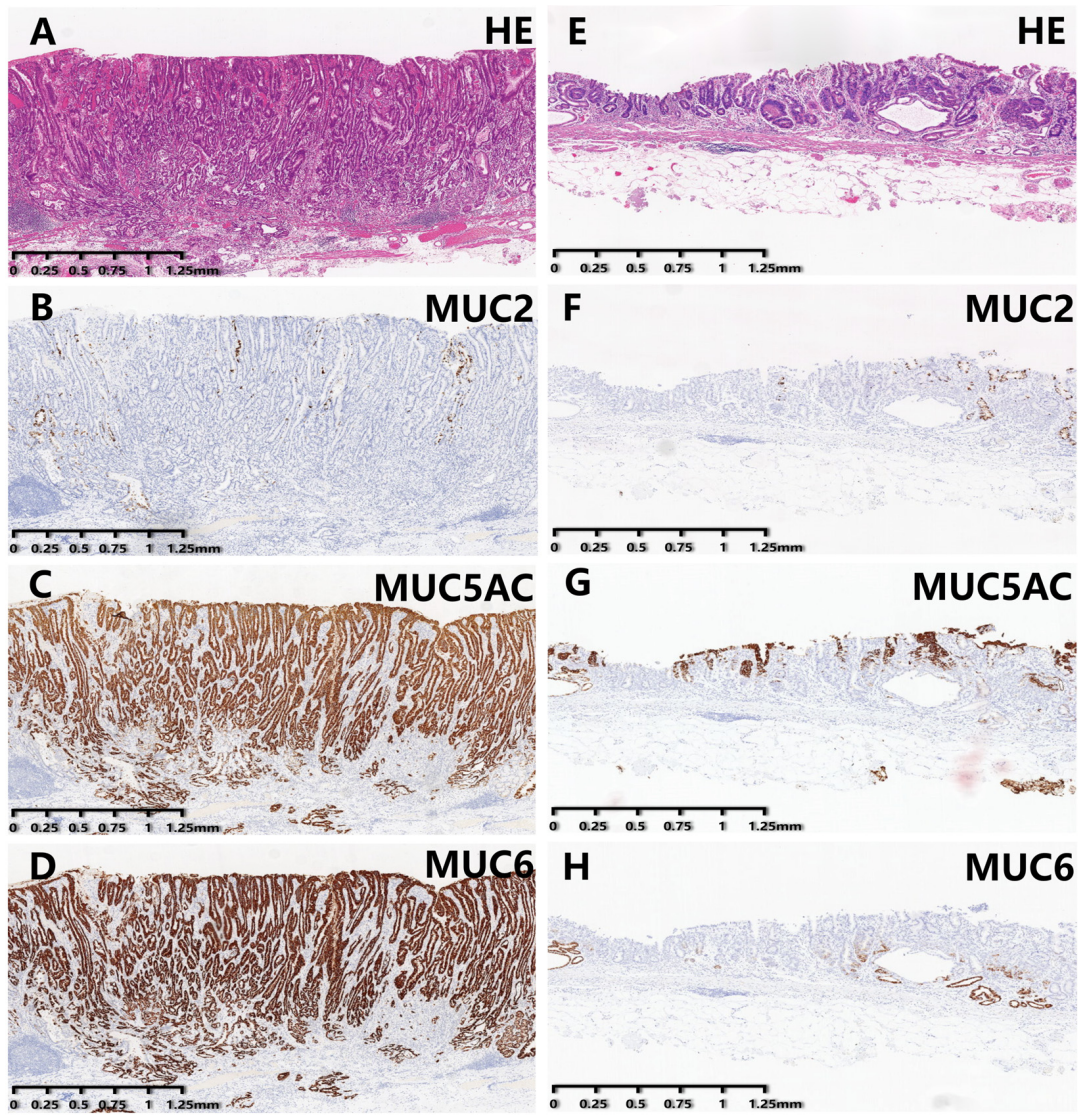
Immunohistochemical staining results of EGPA and EGDTA cases. **(A–D)** HE staining and expression level of mucin in EGPA cases. **(E–H)** HE staining and expression level of mucin in EGDTA cases. Expression level of MUC5AC and MUC6 in EGPA cases was significantly higher than that in EGDTA, while MUC2 expression was comparable between the two groups.

As summarized in **Table 1**, gender, presence of ulceration, infiltration pattern, and MUC2 expression were comparable between the two groups. Compared with EGDTA, EGPA was predominantly located in the lower stomach (*χ*²=6.39, *P*=0.042). At the same time, EGPA mostly manifested as a uniform (*χ*²=47.20, *P*<0.001) and elevated (*χ*²=26.83, *P*<0.001) pattern under endoscopic examination, with VEC structural positivity (*χ*²=104.79, *P*<0.001) observed under ME-NBI. In addition, the tumor diameter of EGPA was larger, with a median diameter of 2.25 cm. Importantly, EGPA was more prone to infiltrate the submucosal layer, contain poorly differentiated cancer components, and invade lymphatic and blood vessels. Finally, EGPA was characterized by high expression levels of gastric-type mucins, namely MUC5AC and MUC6 (**Figure 2**).

### 3.2 Independent distinguishing factors between EGPA and EGDTA

**Figure 3:**
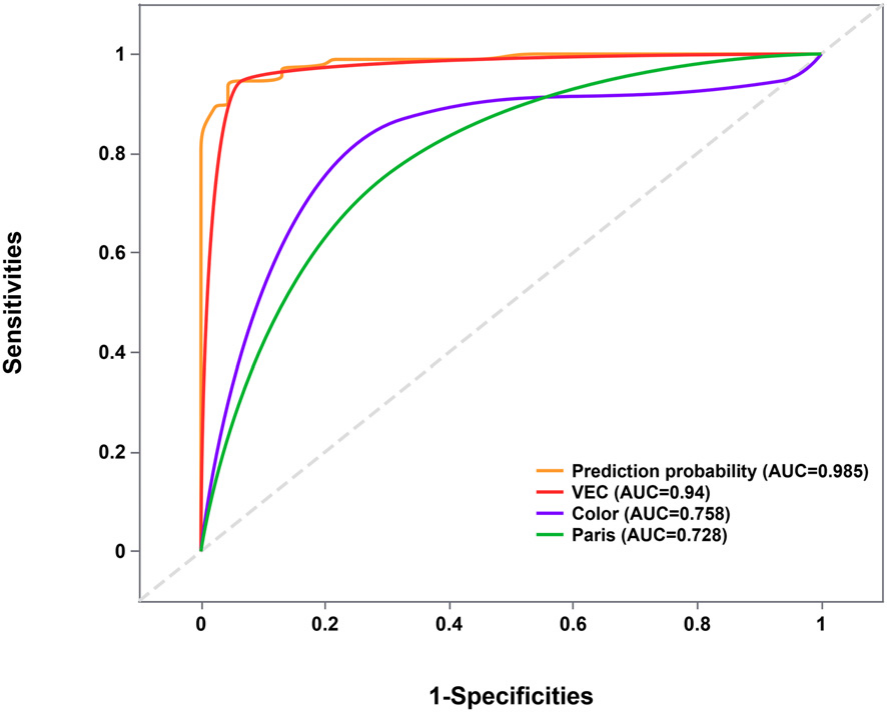
ROC curve evaluating the joint predictive probability of the three factors. ROC, Receiver Operating Characteristic.

To identify distinguishing endoscopic features between EGPA and EGDTA, significant endoscopic features from the univariate analysis were introduced into the multivariate analysis. As detailed in **Table 2**, elevated subtypes, similar coloration to adjacent healthy tissues, and the presence of VEC structures were identified as predictive factors for papillary adenocarcinoma glandular structures. Next, the diagnostic performance of these factors was evaluated using ROC curves, which yielded an area under the curve of 0.985 (95%CI 0.970-1.000, *P*<0.001), with a sensitivity of 95.7% and specificity of 93.5% (**Figure 3**).

**Table 2:** Independent distinguishing factors between EGPA and EGDTA.

Variables	*β*	Standard error	*Wald*	*P*	OR (95%CI)
Tumor location					
Upper					1.00 (Reference)
Middle	-1.21	1.45	0.70	0.40	0.30 (0.02 - 5.12)
Lower	-0.73	1.40	0.27	0.60	0.48 (0.03 - 7.45)
General type					
Non-elevated type					1.00 (Reference)
Elevated type	2.52	1.14	4.87	0.03	12.45 (1.33 - 117.02)
Color					
Red					1.00 (Reference)
Equal	2.12	1.05	4.11	0.04	8.34 (1.07 - 64.82)
Faded	0.73	1.40	0.27	0.60	0.48 (0.31 - 7.45)
VEC					
Negative					1.00 (Reference)
Positive	5.96	1.17	26.11	<0.001	388.26 (39.44 - 3821.68)

### 3.3 Relationship between VEC structure and the degree of malignancy of EGPA

The glandular interspace was used to assess the size of the VEC structure. A total of 43 EGPA patients had positive VEC structures and were divided into two subgroups based on the presence of poorly differentiated components, as well as into mucosa and submucosa subgroups based on infiltration depth. As anticipated, univariate analysis demonstrated that glandular interspace was correlated with the malignancy degree of EGPA. Specifically, EGPA cases with poorly differentiated components had a median glandular interspace value of 74.5, which was smaller than that of cases without poorly differentiated components (**Table 3**). Likewise, EGPA patients with submucosal infiltration had a median glandular interspace value of 82.2, which was smaller than the value of cases with mucosal infiltration (**Table 4**).

**Table 3:** The relationship between the merging of low-differentiated components and VEC structure size in EGPA.

Parameter	With poor differentiation (n=10)	Without poor differentiation (n=33)	*Z*	*P*
glandular interspace (µm)	74.5 (61.6, 93.0)	138.0 (121.8, 153.3)	3.306	< .001

**Table 4:** The relationship between EGPA infiltration depth and VEC structure size.

Parameter	Submucosa (n=8)	Mucosa (n=35)	*Z*	*P*
glandular interspace (µm)	82.2 (59.2, 135.5)	136.3 (95.5, 153.0)	2.122	0.034

### 3.4 ROC curve analysis

**Figure 4:**
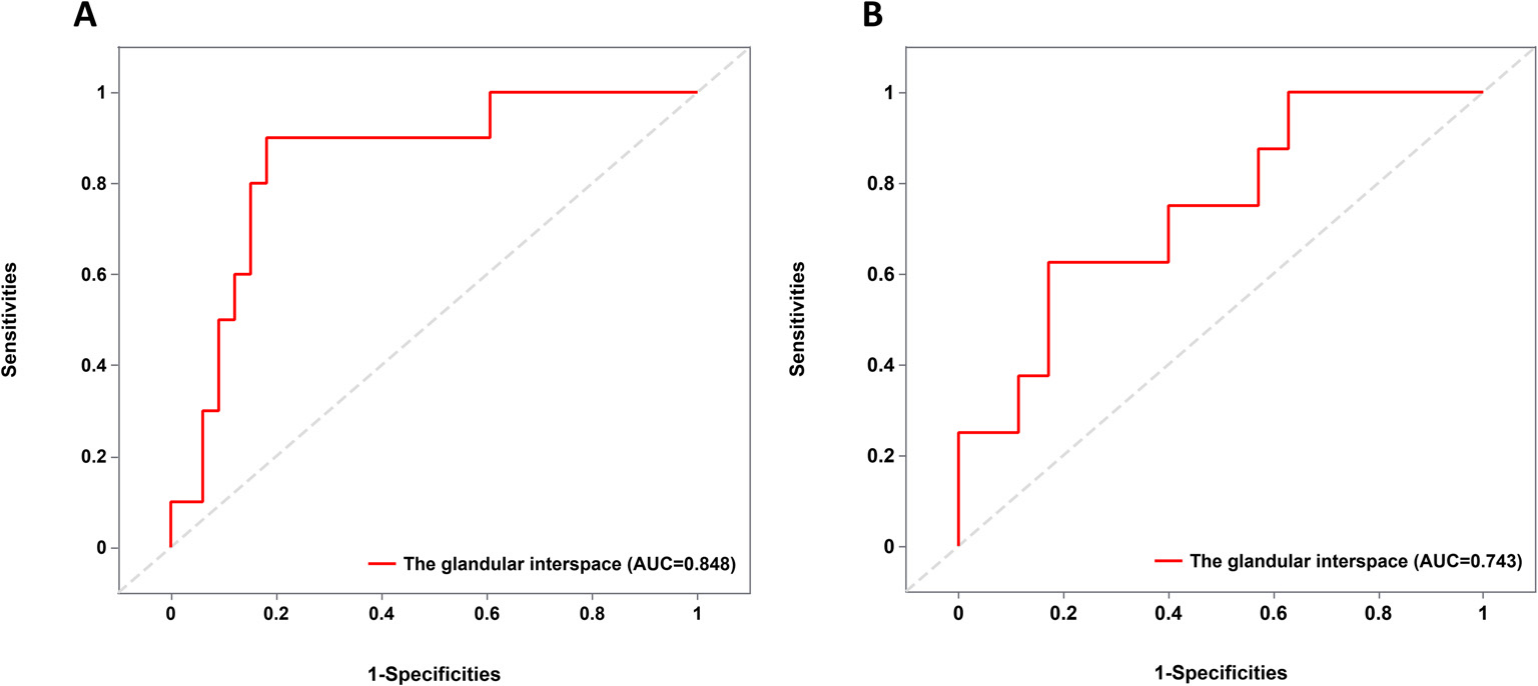
ROC curve analysis of cutoff value. **(A)** Cut-off value for glandular interspace to predict the presence of poorly differentiated carcinoma components in EGPA. **(B)** Cut-off value for glandular interspace to predict the submucosal infiltration in EGPA. ROC, Receiver Operating Characteristic; EGPA, Early Gastric Papillary Adenocarcinoma.

As illustrated in **Figure 4**, a glandular interspace of ≤ 105 μm was identified as the cutoff value for predicting the co-existence of poorly differentiated carcinoma components in EGPA cases. In contrast, a glandular interspace of ≤ 85 μm was identified as the cutoff for predicting submucosal infiltration in EGPA patients.

## 4 Discussion

The fifth edition of the World Health Organization’s classification of digestive tract tumors (19) categorizes gastric adenocarcinoma into tubular adenocarcinoma, papillary adenocarcinoma, mucinous adenocarcinoma, poorly cohesive carcinoma, and other rare histological subtypes. A previous study described that the histopathological features of gastric papillary adenocarcinoma are finger-like protrusions on the surface of axis fibrous blood vessels covered with columnar or cuboidal epithelial cells (7). Of note, it has a low incidence rate and is considered a well-differentiated adenocarcinoma with low cellular pleomorphism. However, studies have concluded that GPA has higher rates of vascular invasion, lymph node metastasis, and liver metastasis compared to differentiated tubular adenocarcinoma of the stomach (20, 21). Additionally, patients with gastric papillary adenocarcinoma have lower postoperative survival rates and shorter life expectancy (22), which garnered extensive attention from gastroenterologists and pathologists. The results of the differential analysis of the pathological characteristics between EGPA and EGDTA herein are in line with the aforementioned finding. Our results collectively unveiled that early gastric papillary adenocarcinoma is more likely to present with submucosal infiltration, accompanied by poorly differentiated carcinoma components and vascular invasion. Therefore, we postulate that it is associated with a higher degree of malignancy and a worse prognosis than early gastric differentiated tubular adenocarcinoma. Ascribed to the rarity of EGPA and the low proportion of papillary adenocarcinoma glands in cancer tissues, biopsy at common sites are prone to misdiagnosis. Therefore, exploring the characteristic endoscopic manifestations of EGPA and correlation with malignancy is significant in guiding the selection of biopsy sites and individualized treatment methods.

At present, endoscopy remains the key method for diagnosing and differentiating gastric cancer. Alterations in mucosal color, gross appearance, and presence of ulcers using endoscopy and visualizing the microstructure of the gastric mucosa surface using ME-NBI contributes to the timely and accurate diagnosis of gastric cancer (23, 24). Lesion color is related to the distribution of blood vessels. Differentiated gastric cancer is accompanied by stromal tissue angiogenesis, resulting in a red mucosal hue. The vessels in the adjacent healthy tissues of undifferentiated carcinoma are more susceptible to damage, resulting in mucosal discoloration (25, 26). Consequently, changes in mucosal color are key to assessing the degree of tumor differentiation. Distinct from other differentiated gastric adenocarcinomas, no significant color difference was noted between EGPA and adjacent healthy tissues during endoscopic examination, attributable to the characteristic structural feature of blood vessels surrounded by white areas, specifically the VEC structure observed under ME-NBI. This study showed that compared to EGDTA, EGPA is more likely to be located in the lower stomach and appear as a homogeneous, elevated type under endoscopy. Furthermore, logistic regression analysis identified elevated subtypes, uniform coloration, and VEC positivity as independent parameters for distinguishing early gastric papillary adenocarcinoma from early gastric differentiated tubular adenocarcinoma under endoscopy, with the three parameters yielding a predictive accuracy of 98.5% for EGPA.

Besides, ROC curve analysis delineated that the VEC structure has a high predictive value for the diagnosis of EGPA (AUC=0.940, 95%CI 0.891-0.990). Therefore, we speculate that the VEC structure is a characteristic feature of EGPA. Interestingly, the three cases without VEC structure all had elevated lesions, with two located in the lower stomach. This finding may be attributed to the inability of ME-NBI to comprehensively observe the anal side of raised lesions (27), leading to a limited field of view and false negatives. In 5 out of the 92 EGDTA cases, while VEC structures were observed, papillary carcinoma components were absent. The VEC structure corresponds to non-tumorous vascular repair hyperplasia and appears as a nipple-shaped structure.

In clinical practice, a potential correlation was identified between the size of the VEC structure and EGPA severity. By correlating endoscopic images with pathological tissue sections, the circular white portion of the VEC structure was found to correspond to the interstitial component with nipple-like structures, whilst microvessels within the white circle to the blood vessels in the ductal protrusions of papillary adenocarcinoma. Furthermore, the inter-glandular spacing was measured in digital pathology tissue sections to reflect the size of VEC structures and analyze the relationship between VEC structure size and the presence of poorly differentiated cancer components as well as submucosal infiltration. Our results exposed that smaller VEC structures were associated with a higher risk of low-differentiated cancer components and submucosal infiltration. Therefore, a raised lesion with no difference in color from the surrounding normal mucosa and the presence of VEC structure should be further evaluated using ME-NBI. If a VEC structure is detected, the size of papillary structures should be compared, and a smaller structure location is recommended for biopsy sampling to obtain a more accurate pathological diagnosis. Given the low incidence of EGPA and the limited number of cases with poorly differentiated carcinoma components and submucosal infiltration, multi-center, large-scale studies are necessitated to investigate the relationship between VEC structure and EGPA severity, as well as identify diagnostic cutoff values to optimize biopsy site selection and treatment strategies.

Meanwhile, this study analyzed differences in mucin protein expression between EGPA and EGDTA patients using immunohistochemical staining, and our results showed that while the expression level of intestinal mucin MUC2 was increased in both groups, the difference was not statistically significant. In contrast, the expression levels of gastric mucins MUC5AC and MUC6 were significantly higher in patients with early gastric papillary adenocarcinoma and were lower or absent in those with early gastric differentiated tubular adenocarcinoma, indicating that MUC5AC and MUC6 may serve as discriminatory biomarkers for EGPA and EGDTA. Research has shown that gastric mucin is associated with the invasive biological behavior of gastric cancer, which may contribute to the poor prognosis of EGPA patients (28). It is worthwhile acknowledging that the degree of co-expression of MUC2, MUC5AC, and MUC6 varies in EGPA patients, indicating a mixed gastrointestinal mucin phenotype.

## 5 Conclusion

This study demonstrated that early gastric papillary adenocarcinoma is linked to a higher degree of malignancy compared to other gastric differentiated adenocarcinoma. Raised lesions with uniform color under endoscopy, accompanied by the presence of a VEC structure under ME-NBI, are highly suggestive of EGPA. Moreover, smaller VEC structures were found to be correlated with a higher degree of EGPA-associated malignancy, offering valuable insights into guide biopsy site selection.

## 6 Limitations

Nevertheless, some limitations of our study that merit acknowledgment. To begin, due to the low incidence of EGPA, the sample size in this study was relatively small. Thus, future multi-center large-scale studies are needed to validate the conclusions of this study. Secondly, the unique expression pattern of mucin suggests a potential germinal origin, possibly associated with gastric foveal epithelial cells, cervical mucous cells, and gastric pyloric gland cells. However, further investigation is required to elucidate the underlying mechanism.

## Data Availability

The raw data supporting the conclusions of this article will be made available by the authors, without undue reservation.
